# Construction and improvement of English vocabulary learning model integrating spiking neural network and convolutional long short-term memory algorithm

**DOI:** 10.1371/journal.pone.0299425

**Published:** 2024-03-22

**Authors:** Yunxia Wang

**Affiliations:** Nanyang Medical College, Nanyang, Henan, China; Menoufia University, EGYPT

## Abstract

To help non-native English speakers quickly master English vocabulary, and improve reading, writing, listening and speaking skills, and communication skills, this study designs, constructs, and improves an English vocabulary learning model that integrates Spiking Neural Network (SNN) and Convolutional Long Short-Term Memory (Conv LSTM) algorithms. The fusion of SNN and Conv LSTM algorithm can fully utilize the advantages of SNN in processing temporal information and Conv LSTM in sequence data modeling, and implement a fusion model that performs well in English vocabulary learning. By adding information transfer and interaction modules, the feature learning and the timing information processing are optimized to improve the vocabulary learning ability of the model in different text contents. The training set used in this study is an open data set from the WordNet and Oxford English Corpus data corpora. The model is presented as a computer program and applied to an English learning application program, an online vocabulary learning platform, or a language education software. The experiment will use the open data set to generate a test set with text volume ranging from 100 to 4000. The performance indicators of the proposed fusion model are compared with those of five traditional models and applied to the latest vocabulary exercises. From the perspective of learners, 10 kinds of model accuracy, loss, polysemy processing accuracy, training time, syntactic structure capturing accuracy, vocabulary coverage, F1-score, context understanding accuracy, word sense disambiguation accuracy, and word order relation processing accuracy are considered. The experimental results reveal that the performance of the fusion model is better under different text sizes. In the range of 100–400 text volume, the accuracy is 0.75–0.77, the loss is less than 0.45, the F1-score is greater than 0.75, the training time is within 300s, and the other performance indicators are more than 65%; In the range of 500–1000 text volume, the accuracy is 0.81–0.83, the loss is not more than 0.40, the F1-score is not less than 0.78, the training time is within 400s, and the other performance indicators are above 70%; In the range of 1500–3000 text volume, the accuracy is 0.82–0.84, the loss is less than 0.28, the F1-score is not less than 0.78, the training time is within 600s, and the remaining performance indicators are higher than 70%. The fusion model can adapt to various types of questions in practical application. After the evaluation of professional teachers, the average scores of the choice, filling-in-the-blank, spelling, matching, exercises, and synonyms are 85.72, 89.45, 80.31, 92.15, 87.62, and 78.94, which are much higher than other traditional models. This shows that as text volume increases, the performance of the fusion model is gradually improved, indicating higher accuracy and lower loss. At the same time, in practical application, the fusion model proposed in this study has a good effect on English learning tasks and offers greater benefits for people unfamiliar with English vocabulary structure, grammar, and question types. This study aims to provide efficient and accurate natural language processing tools to help non-native English speakers understand and apply language more easily, and improve English vocabulary learning and comprehension.

## Introduction

As a second language used worldwide, English is of great significance to many non-native English speakers. Mastering English vocabulary is the foundation of language learning, and it is crucial to improve the ability to speak, read, and write. However, the process of learning English vocabulary is often challenging, especially for non-native English speakers [1]. With the rapid development of artificial intelligence (AI) and machine learning, researchers begin to apply these technologies to construct and improve English vocabulary learning models. Spiking Neural Network (SNN) is a new neural network (NN) model, which mimics the pulse transmission mode between neurons in the human brain, and has high biological interpretability and computational efficiency. The Convolutional Long Short-Term Memory (Conv LSTM) algorithm is a deep learning (DL) model that combines Convolutional Neural Network (CNN) and Long Short-Term Memory (LSTM), with the ability to process sequence data and image data [2].

This study aims to explore the application of SNN and Conv LSTM algorithms to the construction and improvement of English vocabulary learning models. Firstly, the existing vocabulary learning model is analyzed, and the existing problems and challenges are put forward. Secondly, the principles and characteristics of SNN and Conv LSTM algorithms are studied, and then an English vocabulary learning model integrating SNN and Conv LSTM algorithms is designed and implemented. Finally, the effectiveness of the optimized model in this study is verified by experiments. The results of this study are expected to provide a novel and efficient English vocabulary learning model for non-native English speakers. Meanwhile, this study also helps to promote the application and development of SNN and DL algorithms in the field of education, opening up new possibilities for the combination of language education and AI technology.

## Literature review

In traditional research, there have been some studies on constructing and improving English vocabulary learning models. Mandasari and Wahyudin (2021) explored the impact of online contextualization on English vocabulary learning. The results showed that combining vocabulary learning materials and actual context can improve the learner’s vocabulary learning effect. However, this study mainly relied on manual annotation and text processing techniques and lacked the exploration of using the DL algorithm to improve efficiency and accuracy [3]. Zhang et al. (2020) studied the effect of enhancing English vocabulary learning through word association. They found that word association can help learners to remember and use new words better. However, this study had yet to fully utilize the DL algorithm’s potential, and only adopted traditional association technology [4]. Maqsood et al. (2022) comprehensively assessed computer-aided (CA) vocabulary learning. The results denoted that CA learning tools positively improved learners’ vocabulary and vocabulary mastery. However, studies also pointed out some limitations of existing CA learning tools, such as inadequate adaptability to individual differences and insufficient guidance on learning strategies [5]. Chen and Hsu (2020) evaluated the effect of mobile device-assisted vocabulary learning through a meta-analysis of a large number of relevant studies. They found that in the mobile learning environment, learners learned vocabulary using mobile applications and achieved significant learning results. But the study also proposed the challenges of existing mobile-assisted learning methods in adapting to individual needs and creating interactive learning environments [6]. In the study of NNs, Turukame et al. (2022) reviewed the monitoring methods of DL classification techniques. Studies revealed that employing a secure location protocol can improve the recognition performance of NNs in monitoring systems, but the training time was still a problem [7]. Wongchai et al. (2022) improved the monitoring performance of the DL model by adjusting the neural network architecture, changing the position of neurons in the forgetting gate of the model, and enhancing the neural networks’ monitoring performance. However, the problem that the sample size of each input of the model was too small was still not solved [8].

To sum up, traditional research on English vocabulary learning revealed the positive effects of CA, mobile, contextualized, gamified, and multimedia annotation on vocabulary learning. Nevertheless, these approaches faced challenges and limitations, such as inadequate personal adaptability, learner motivation issues, and balancing the relationship between learning and entertainment. In contrast, the research method in this study integrated SNN and Conv LSTM algorithms into the English vocabulary learning model to improve the personalized adaptability, advanced sequence processing ability, and accuracy of the model effect, which provided better learning support and improved the expression ability of non-native English learners.

## The application of SNN and Conv LSTM algorithms in English vocabulary learning model

### Problems existing in traditional English vocabulary learning model

Traditional English vocabulary learning methods have always been an important way for learners to master and remember English words. These traditional methods are mainly exhibited in Table 1.

**Table 1:** Traditional English vocabulary learning methods.

Learning methods	Specific advantages and disadvantages
Memory-based learning	This method emphasizes mastering English words through repeated memorization. Learners repeatedly display and memorize words to understand and apply them in practical situations. Although this method can help learners remember some words in the short term, long-term memory and application effect are limited, and lack systematicness and creativity, so it is difficult to cope with the learning needs of many words.
Context-based learning	This method learns by placing words in specific sentences or contexts. Learners understand words by observing their usage and meaning in different sentences. For example, vocabulary can be learned by reading articles, listening to conversations, or watching movies. This method provides certain contextual information, but its scope is limited and requires many example sentences and corpus support to cover a wider range of contexts.
Learning based on root and affix	This method focuses on the role of roots and affixes in word formation and meaning. Learners infer the meaning and usage of words by analyzing and understanding their structure and components. For instance, learners can infer the meaning of biology from the root word "bio" and the affix "logic". However, this method has certain requirements for learners’ vocabulary foundation and linguistic knowledge, requiring a longer learning and accumulation period.
Learning based on association and memory substitution	This method memorizes new words by associating them with known things or concepts. Learners attempt to associate new words with existing knowledge, experience, or situations to enhance memory performance. For example, connecting the word ’apple’ with the actual fruit image can help remember the spelling and meaning of the word.

The advantage of traditional methods is that they are easy to understand and implement and can be adapted and extended according to the needs of learners. However, they are often not systematic and personalized, and cannot be well adapted to learners’ characteristics and learning goals [9–12]. In addition, due to the large and varied vocabulary, the traditional model is difficult to cover all words, and it is not easy to cope with the diversity of meanings and the complexity of contexts.

### Application of SNN and Conv LSTM algorithms in information processing

SNN is a simulation algorithm based on the biological neural network (BNN), which plays an important role in timing information processing and pattern recognition tasks. Unlike the traditional NN model, SNN represents the communication between neurons with discrete events, and transmits and processes information through time interval and frequency coding [13].

SNN has the following advantages in timing information processing. The first is time accuracy. SNN can more accurately capture and process the time correlation in the input data. Since the occurrence time and frequency of pulses play a key role in information encoding, SNN can more accurately perceive and respond to subtle patterns and dynamic changes. The second is energy efficiency. Compared with traditional NN models, SNN consumes less computing resources and energy when processing timing information. This is because the SNN is only activated when a pulse is received and is computed sparsely in time, reducing computation overhead and power consumption. The last is asynchronous communication. Communication between neurons in SNN is carried out asynchronously. That is, neurons autonomously generate pulses based on their internal state and transmit information to other neurons via the pulses. This allows SNN to process parallel and distributed timing information, helping to simulate complex time-dependent tasks in the real world.

Conv LSTM algorithm is a model that combines CNN and LSTM, which is of great significance for sequence data modeling task. Compared with traditional Recurrent Neural Network (RNN) and LSTM models, Conv LSTM takes advantage of convolutional operations’ local connectivity and weight sharing characteristics and can also process and remember long-distance dependencies in sequences [14–17]. In sequence data modeling, its advantages are outlined in Table 2.

**Table 2:** Advantages of the Conv LSTM algorithm in sequence data modeling.

Advantages	Concrete content
Context information modeling	Conv LSTM can automatically learn and capture contextual information in input data. Through convolution operation, Conv LSTM can detect local patterns in space at different time steps, and store and transmit this information in the gating mechanism and memory unit of LSTM. This enables Conv LSTM to understand better and model sequence data’s overall semantics and structure.
Parameter sharing and computational efficiency	Conv LSTM reduces the number of parameters that need to be learned through weight sharing and local connectivity, and can perform calculations efficiently. This makes Conv LSTM more efficient in processing large-scale sequence data and able to cope with complex spatiotemporal relationships in practical applications.
Handling long-distance dependencies	The RNN model is prone to gradient vanishing or exploding when processing long sequence data, making it difficult to capture long-distance dependencies. Conv LSTM, by combining convolution and LSTM structures, can better handle long-term dependencies in sequences, avoiding the limitations of traditional RNN models.
Parallelization operation	Due to the local connectivity of convolution operations in Conv LSTM, this algorithm can effectively parallelize computation to accelerate the training process. This parallelization capability gives Conv LSTM an advantage in processing sequence data with many time steps, and is suitable for efficient hardware accelerator implementation.

Conv LSTM has been extensively used in many fields, such as video analysis, motion recognition, natural language processing (NLP), etc. It can effectively model the spatio-temporal relationship in sequence data and provide more accurate prediction and analysis results [18–21]. Moreover, Conv LSTM is also used for image generation, video prediction, and other tasks, demonstrating its powerful representation and modeling capabilities for sequence data.

### Establishment of the English vocabulary learning model based on SNN and Conv LSTM algorithms

SNN is a biologically inspired NN model, which simulates the working mechanism of brain neurons and represents the information transmission between neurons through discrete pulse signals [22]. Neuronal models in SNN are usually based on the Izhikevich model of pulse conduction or the Leaky integrand-fire model. These models can generate a pulsed output based on the input current and the neuron’s membrane potential. SNN uses a time-encoding scheme to represent information [23]. While traditional NNs perform calculations based on the level of neuron activation, SNN encodes information by the arrival time and frequency of the pulses. Higher frequencies indicate stronger activation. When a neuron’s membrane potential exceeds a threshold, it generates a pulse and transmits it to the connected neuron. In this way, the transmission of information between neurons is carried out through discrete pulse signals.

**Fig 1:**
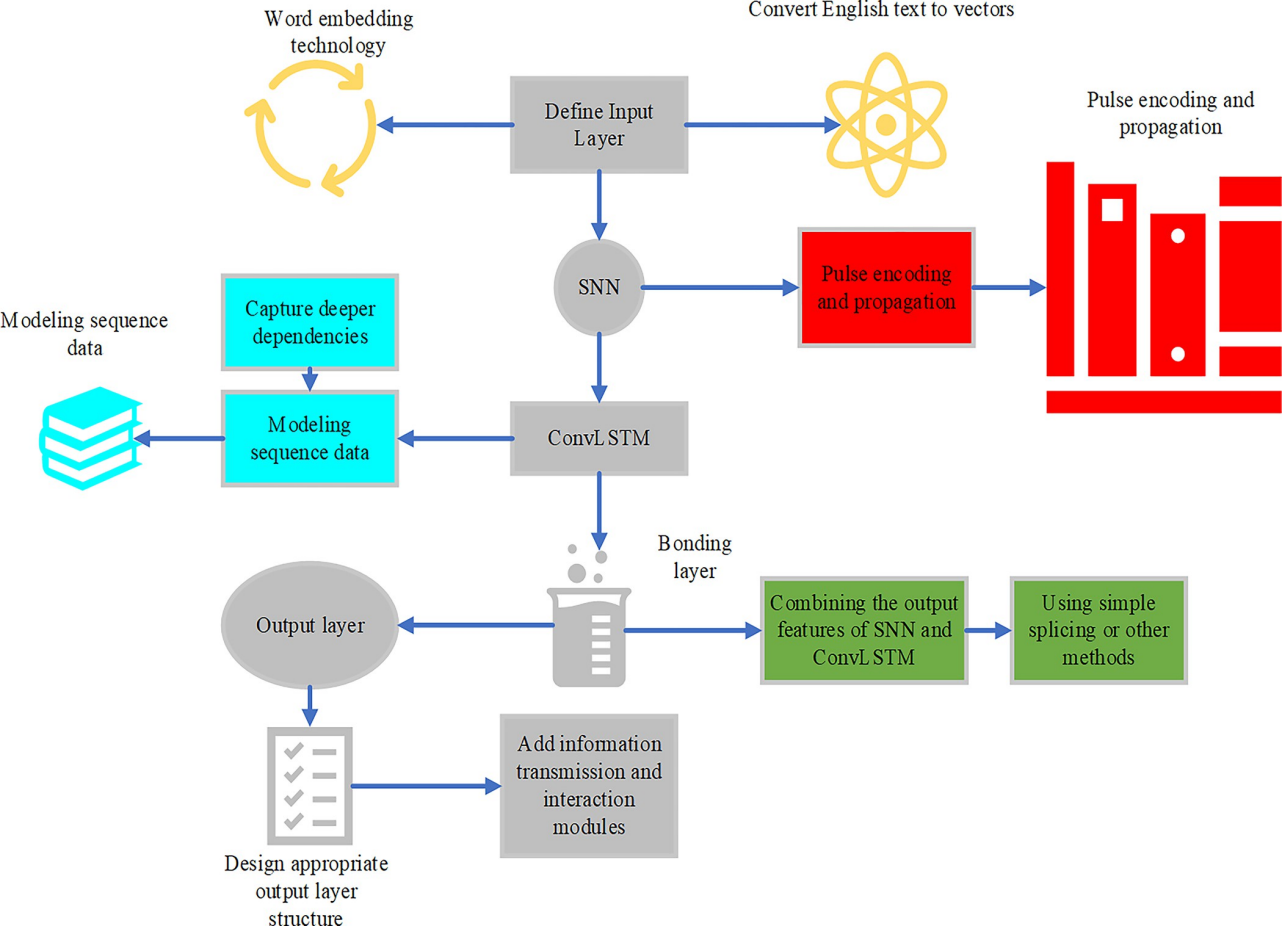
English vocabulary learning model by the SNN and Conv LSTM algorithms.

The model structure of Conv LSTM consists of a series of Conv LSTM units. Each Conv LSTM unit includes an input gate, a forget gate, and an output gate, as well as a cell state and a hidden state [24–26]. Different from traditional LSTM, the input, forget, and output gates of Conv LSTM, as well as cell and hidden states, are all two-dimensional matrices [27]. Therefore, this study integrates SNN and Conv LSTM algorithms, and adds information transfer and interaction modules to improve the effect of English vocabulary learning. The new model is displayed in Fig 1.

Using SNN to process temporal information can better capture temporal correlations and long-term dependencies in temporal data than traditional NN models. This makes SNN potentially useful in processing time-related tasks such as speech recognition, time series prediction, and brain signal analysis. The merit of Conv LSTM in processing sequence data is that it can consider both spatial and temporal features, and effectively process image sequence and video data. Conv LSTM has more advantages than traditional LSTM models regarding computational efficiency and modeling capability. This makes Conv LSTM widely used in image timing prediction, video frame interpolation, action recognition, and other tasks.

By integrating SNN and Conv LSTM algorithms, and adding information transfer and interaction modules, the model’s time series modeling ability and feature extraction ability can be further improved. Such a fusion model can exhibit better performance and effect in tasks dealing with timing information, such as text generation, speech recognition, video analysis, etc.

### Experimental design

The data sets used here are the public data sets in WordNet and Oxford English Corpus. WordNet is an English dictionary and vocabulary database, which organizes a lot of words and related information about their meanings based on the concept of words. WordNet organizes words into a network of meanings in which words and their various meanings are called "synsets". Each synset represents a word’s meaning; multiple words can be associated with a synset. It divides the relationship between the upper and lower words among different meanings. WordNet data set can be accessed through the official website to obtain data: https://wordnet.princeton.edu/. The Oxford English Corpus is a large and diverse corpus containing massive English text samples from diverse textual sources for the study and analysis of language use in English. It collects samples of texts from different fields and genres, involving books, blogs, newspapers, journals, social media, and more. The samples come from English speakers in different countries and regions, covering a variety of language styles and styles. The Oxford English Corpus data set can be accessed through the official website to obtain data: https://ota.bodleian.ox.ac.uk/repository/xmlui/handle/20.500.12024/2554.

In the experimental environment, the Central Processing Unit (CPU) size is 2.5G, the server’s system version is Windows 7, the hard disk size is 12GB, and the network is Apache-tomecat6. Part of the code used to run the model in the experiment is as follows:

#Add embedded layer
Model. add (Embedding (input_dim = vocab_size, output_dim = embedding_dim, input_length = sequence_length))
#Add one-dimensional convolutional layer
Model. add (Conv1D (filters = num_filters, kernel_size = filter_size, activation = ’redu ’)
Model. add (Dropout (rate = 0.2))

The experiment needs to unify the parameters, where the LSTM quantity is 16, the learning rate is 0.001, the regularization parameter is 8, the batch size is 16, and the activation function is Relu.

## Performance analysis of English vocabulary learning model using SNN and Conv LSTM algorithms

### Performance analysis of English vocabulary learning model after optimization

**Fig 2:**
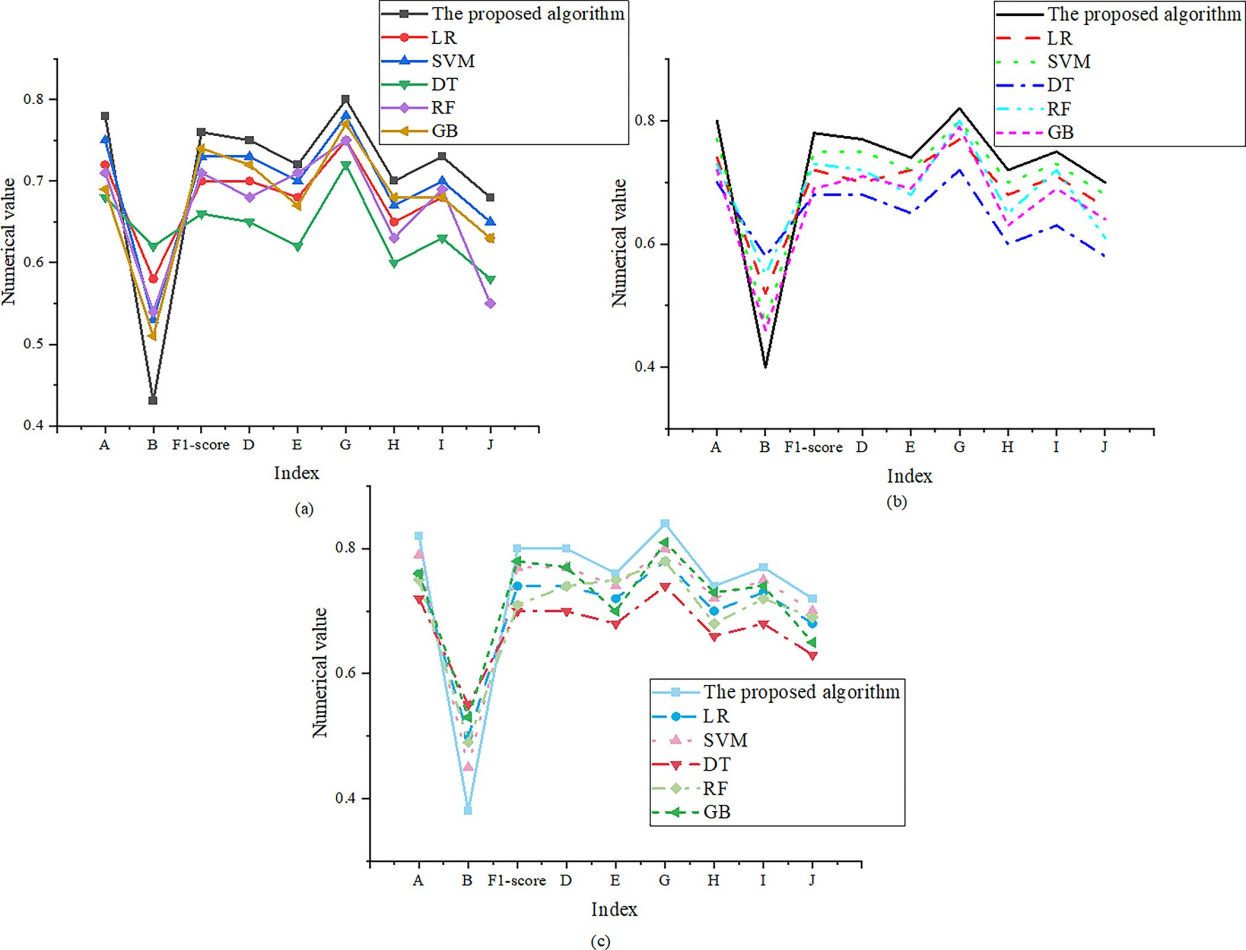
Comparison results of model performance under the 100–400 training set: ((a), (b), and (c) represent that the size of the training set is 100, 200, and 400).

The optimization model proposed in this study is compared with five traditional models. The comparison models are Logistic Regression (LR), Support Vector Machine (SVM), Decision Tree (DT), Random Forest (RF), and Gradient Boosting (GB). From the learners’ viewpoint, the model’s accuracy, loss, F1-score, training time, polysemy processing accuracy, syntactic structure capturing accuracy, vocabulary coverage, context understanding accuracy, word sense disambiguation accuracy, and word order relation processing accuracy are considered. For the convenience of the experiment, A refers to accuracy; B stands for loss; D means polysemy processing accuracy; E represents syntactic structure capture accuracy; G indicates word coverage; H denotes context understanding accuracy; I displays word sense disambiguation accuracy; J signifies word order relation processing accuracy. When the training set size is 100, 200, and 400, the model comparison is presented in Fig 2.

In Fig 2, the proposed optimization model’s accuracy is 0.75%-0.77 in the 100–400 text volume range, which means the model can accurately predict text classification. In addition, the model’s loss value is less than 0.45, indicating that the model has a high degree of fitting to the training data. The F1-score is more than 0.75, illustrating that the model can balance accuracy and recall rate to achieve a better prediction effect. The comparison results of training time are plotted in Table 3.

**Table 3:** Comparison of model training time in 100–400 training set.

The size of training sets	The proposed algorithm model	LR	SVM	DT	RF	GB
100	90s	100s	95s	103s	110s	98s
200	186s	210s	195s	203s	199s	198s
400	276s	299s	304s	310s	280s	295s

**Fig 3:**
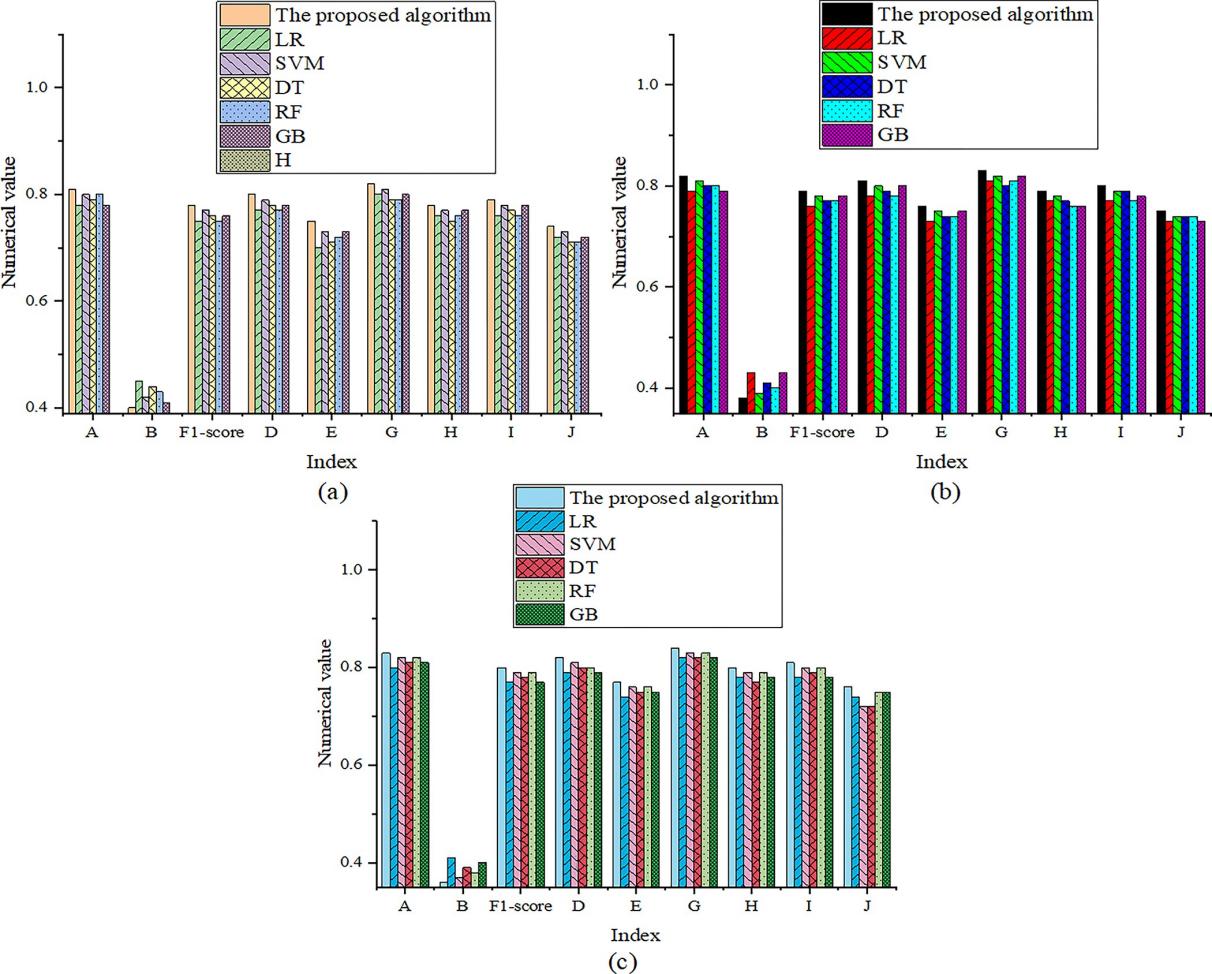
Comparison of model performance in the 500–1000 training set: ((a), (b), and (c) indicate that the training set sizes are 500, 800, and 1000).

Table 3 describes that the training time of the proposed algorithm model is less than 300 seconds, which indicates that the model’s training speed is relatively fast. Besides, the model also achieves good results on other performance indicators, exceeding the 65% level. When the training set size is 500–1000, the performance comparison results of the model are suggested in Fig 3.

According to further analysis of Fig 3, it can be observed that in the range of 500–1000 text volume, the accuracy of the proposed model is 0.81–0.83, the loss is no more than 0.40, and the F1-score is no less than 0.78. The model has reached a high level in all indicators showing its strong ability and comprehensive performance. It means that the proposed model fits the training data well, and the comparison results of training time are revealed in Table 4.

**Table 4:** Comparison of model training time under 500–1000 training set.

The size of training sets	The proposed algorithm model	LR	SVM	DT	RF	GB
500	259s	283s	266s	292s	278s	293s
800	322s	349s	330s	367s	356s	360s
1000	381s	422s	397s	399s	415s	411s

**Fig 4:**
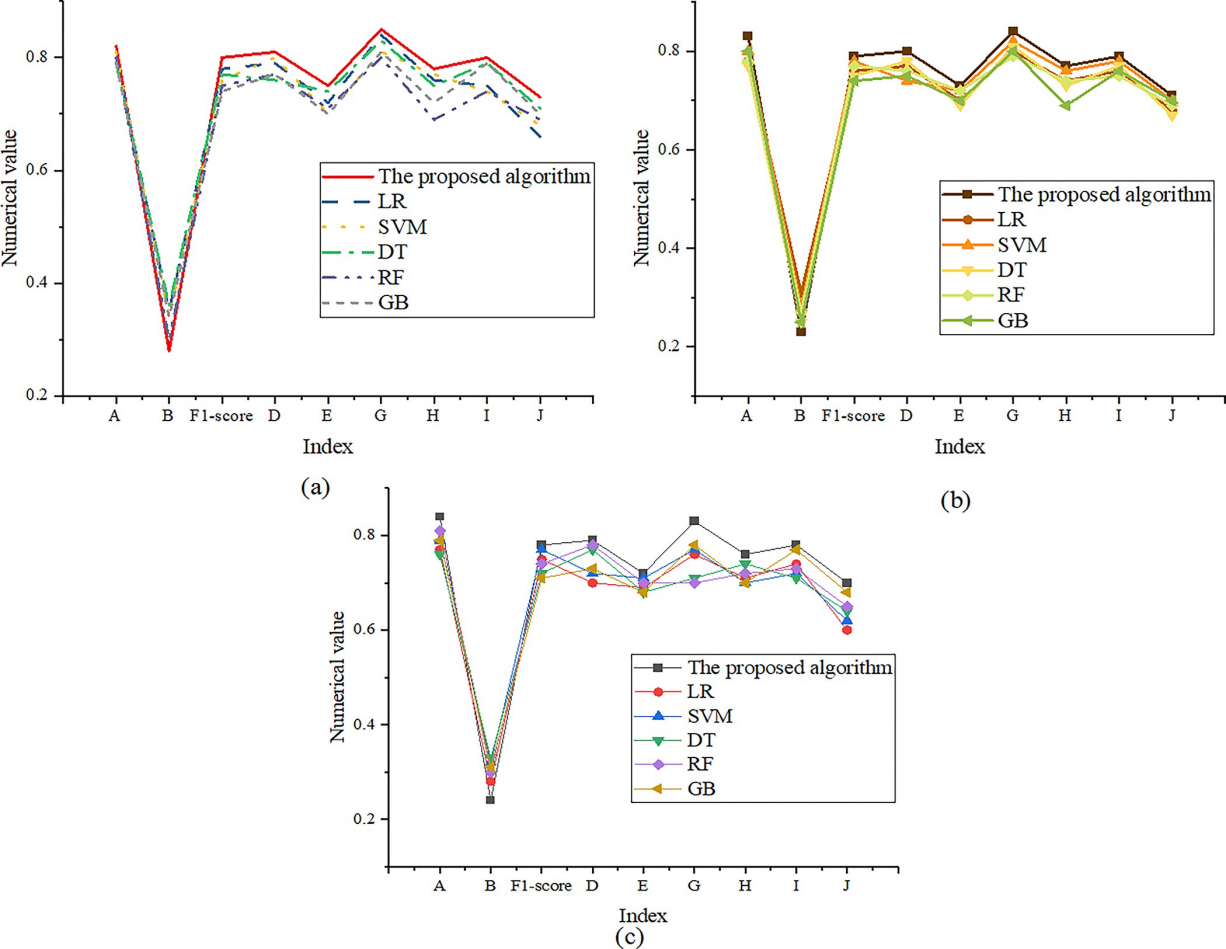
Comparison results of model performance under the 1500–3000 training set (a): The training set size is 1500; (b): The training set size is 2000; (c): The training set size is 3000.

Table 4 demonstrates that the proposed model’s training time is within 400s, which indicates that the model has high training efficiency, enabling it to learn and adapt quickly on large-scale data sets, and other performance indicators are over 70%. When the training set size is 1500–3000, the models’ performance comparison results are expressed in Fig 4.

Fig 4 details that the optimized model performs excellently in the text volume range of 1500–3000. The model’s accuracy is stable in the range of 0.82 to 0.84, which means that the model can make classification predictions of text with high accuracy. With such a high degree of accuracy, the model’s predictions will be very reliable. At the same time, the model’s loss value can remain low, no more than 0.28, indicating a high degree of model adaptation to the training data. The model’s excellent fit means it can effectively capture patterns and features in the data. In terms of F1-score, the model’s performance exceeds 0.78, demonstrating that it has a good balance between balanced prediction effects and recall rates. This balancing performance helps the model to efficiently process various categories of text and provide accurate classification results. The comparison results of training time are shown in Table 5.

**Table 5:** Comparison of model training time in 1500–3000 training set.

The size of training sets	The proposed algorithm model	LR	SVM	DT	RF	GB
1500	521s	563s	570s	600s	570s	591s
2000	530s	590s	610s	623s	634s	644s
3000	560s	783s	677s	654s	670s	710s

Table 5 expresses that the training time of the proposed model is also quite considerable, and it only takes 600 seconds to complete the training. This means that the model has a high training efficiency, allowing it to learn and adapt to large text data sets relatively quickly. Ultimately, in addition to accuracy, loss, and F1-score, the model also shows excellent performance on other performance indicators, exceeding the 70% level. These indicators may include accuracy, recall, class-specific accuracy, etc., further confirming the model’s overall performance.

### Simulation results of the model

**Fig 5:**
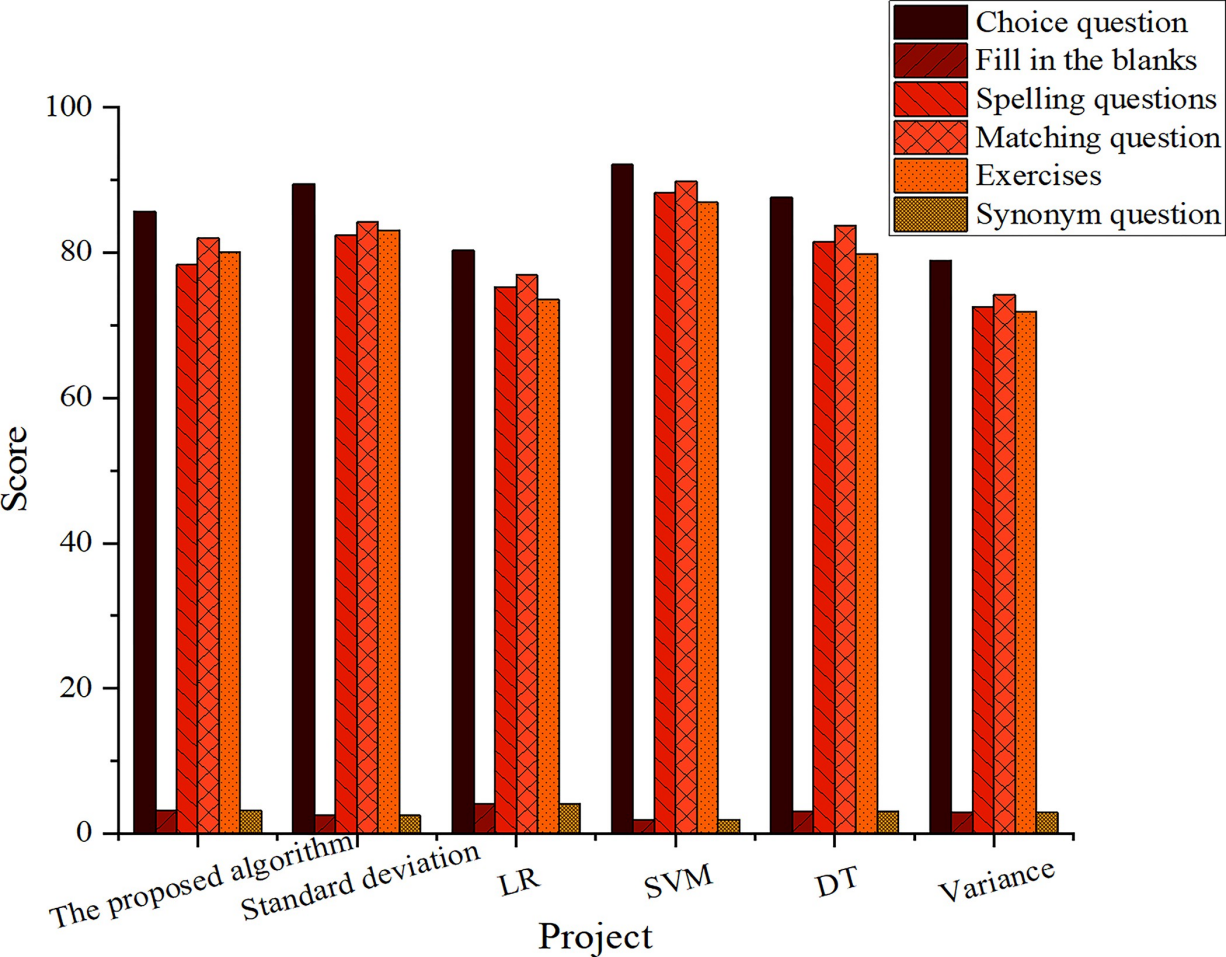
Comparison results of simulation experiments.

Simulation experiments were conducted to verify the proposed model’s feasibility and effectiveness. LR, SVM, and DT models were selected for comparison. Six types of question types were identified and analyzed after evaluation by professional teachers, including choice, fill-in-the-blanks, spelling, matching, exercises, and synonyms. The experimental results are portrayed in Fig 5.

Fig 5 draws that after evaluation by professional teachers, the mean scores of matching, fill-in-the-blank, exercises, choice, spelling, and synonym questions are 92.15, 89.45, 87.62, 85.72, 80.31, and 78.94, respectively, which are far more than other traditional models. It can explain the feasibility and effectiveness of the proposed model. Additionally, combined with the horizontal comparison data of the model, it can be found that the fusion model’s performance is gradually enhanced with the increase of the text volume, showing lower loss and higher accuracy.

## Discussion

According to the experimental results, the performance of the optimized fusion model under different text volumes shows higher superiority. In the range of 100–400 texts, the accuracy of the fusion model is stable between 0.75–0.77, the loss is maintained below 0.45, the F1-score remains above 0.75, the training time is controlled within 300 seconds, and other performance indicators are maintained above 65%. In the range of 500–1000, the fusion model’s accuracy performance is slightly improved, reaching the level of 0.81–0.83, the loss does not exceed 0.40, the F1-score remains more than 0.78, the training time increases to less than 400 seconds, and other performance indicators exceed 70%. When the amount of text is expanded to 1500–3000, the model’s accuracy remains between 0.82–0.84, the loss is significantly reduced to less than 0.28, the F1-score is not less than 0.78, the training time is slightly longer, but it is still controlled within 600 seconds. Other performance indicators are more than 70%. These results manifest that with the rise of text volume, the performance of the fusion model is slowly promoted, reflecting higher accuracy and lower loss. This also shows that this model has strong scalability and generalization ability, and can achieve good performance when dealing with text data of different sizes. The optimized fusion model presents excellent results in English learning tasks in the simulation experiment. According to professional teachers’ evaluation, the fusion model’s average scores in the synonym types, spelling, choice, exercises, filling-in-blank, and matching are 78.94, 80.31, 85.72, 87.62, 89.45, and 92.15. These scores are significantly higher than other traditional models, further verifying the superiority of the fusion model. The study aims to offer accurate and efficient NLP tools to help non-native English speakers understand and apply the language more easily, and improve their English vocabulary learning and comprehension skills. The fusion model’s excellent performance shows its potential and application value, which provides greater help to English learners.

## Conclusion

In the past few years, the swift growth of DL and NLP technologies has provided new opportunities for English vocabulary learning. Here, by combining the strengths of SNN and Conv LSTM algorithms in time series information processing, an English vocabulary learning model combining the two algorithms is designed and constructed. The experimental outcomes show that the performance of the fusion model is better under various text sizes. In the text volume range of 100–400, the fusion model reaches a stable accuracy between 0.75 and 0.77, and a loss control below 0.45. In addition, the F1-score achieves above 0.75. The training time does not exceed 300 seconds, while the other performance indicators are more than 65%. As the number of texts increases to 500–1000, the fusion model’s accuracy improves, between about 0.81–0.83. The loss value remains below 0.40, the F1-score stays above 0.78, with training time limited to 400 seconds or less, while other performance indicators exceed 70%. For the range of 1500–3000, the fusion model still maintains a high accuracy of about 0.82–0.84. The loss is reduced to less than 0.28, while the F1-score is no less than 0.78. Although the training time is slightly longer, it is under 600 seconds. Moreover, other performance indicators are also higher than 70%. Additionally, the fusion model can adapt to various types of questions. 85.72, 89.45, 80.31, 92.15, 87.62, and 78.94 correspond to the average scores on question types such as choice, fill-in-the-blank, spelling, matching, exercises, and synonyms, respectively. These scores are significantly higher than other models, illustrating that the fusion model has a good effect in practical application. This study also has many shortcomings. First, the types of questions covered and the scope of data may be limited, failing to cover all possible situations. Second, the experimental results are based on specific data sets and are limited to English learning tasks, so their applicability may be affected by other tasks and fields. Future studies will be further refined and extended to verify the performance and applicability of the fusion model in a broader range of scenarios.

## Supporting information

S1 Data (ZIP)
