# Supplementary material for: Construction and improvement of English vocabulary learning model integrating spiking neural network and convolutional long short-term memory algorithm
Source: PLoS One. 2024 Mar 22;19(3):e0299425. doi: 10.1371/journal.pone.0299425 (PMC10959372; doi:10.1371/journal.pone.0299425)
Supplement: S1 Data — (ZIP) [file pone.0299425.s001.zip › ╩2╛▌░n/Code Description.docx]

In this code segment, the Keras library's Sequential model is utilized to define the neural network architecture. The Sequential model is a straightforward layered structure that allows for the sequential addition of various layers to the model.

Next, the `model.add()` method is employed to incorporate an Embedding layer into the model. An Embedding layer is used to transform discrete integer input data into a continuous, low-dimensional vector representation. This layer learns the weights associated with these vectors within the neural network. The Embedding layer in this case has three parameters: `input_dim`, which represents the size of the vocabulary (i.e., the number of unique words in the vocabulary); `output_dim`, which signifies the dimension of the output embedding vectors; and `input_length`, which specifies the length of the input sequences.

It is important to note that the values for `vocab_size`, `embedding_dim`, and `sequence_length` mentioned in the code need to be defined and assigned in other parts of the code to ensure the correct construction of the model.

Continuing from this point, the code builds upon the Embedding layer foundation by adding a one-dimensional Convolutional layer and a Bidirectional LSTM layer. A Dropout layer is also inserted between these layers.

Firstly, the code uses the `model.add()` method to add a one-dimensional Convolutional layer to the model. Convolutional layers are commonly used in deep learning to extract features from input data. In this case, the Convolutional layer has three parameters: `filters` representing the number of filters in the layer, `kernel_size` indicating the size of the convolutional kernel, and `activation` specifying the activation function, which is ReLU in this case.

Next, through the `model.add()` method, a Dropout layer is added. The purpose of the Dropout layer is to randomly drop a portion of neurons during network training to reduce the risk of overfitting. The `rate` parameter here specifies the proportion of neurons to drop.

Subsequently, the code again employs the `model.add()` method to introduce a Bidirectional LSTM layer into the model. The Bidirectional LSTM layer considers both forward and backward sequence information at each time step, aiding in capturing contextual information more effectively. The `units` parameter of this layer denotes the number of LSTM units, while `return_sequences=True` signifies that the layer outputs the complete sequence instead of only the output at the last time step.

Lastly, another Dropout layer is added to further mitigate overfitting risks.

Please be aware that prior to running the model, it is essential to ensure that parameters such as `num_filters`, `filter_size`, `lstm_units`, etc., have been defined and assigned.

Continuing from this point, the code proceeds to add a Global Max Pooling layer and a Fully Connected layer after the Bidirectional LSTM layer. Another Dropout layer is inserted once again in the sequence.

Using the `model.add()` method, a Global Max Pooling layer (GlobalMaxPooling1D layer) is added to the model. The purpose of the Global Max Pooling layer is to obtain the maximum value of the features generated by each convolutional kernel, thus obtaining a more representative feature representation. This layer has no parameters.

Following that, through the `model.add()` method, a Fully Connected layer (Dense layer) is added to the model. The Fully Connected layer aggregates all the features from the previous layer, linearly combines them with a set of learnable weights, and applies an activation function. The `units` parameter here specifies the number of neurons in the Fully Connected layer, and the `activation` parameter indicates the activation function, with ReLU being used in this case.

Lastly, another Dropout layer is added once again to further mitigate overfitting risks.

Remember, before running the model, make sure that parameters like `hidden_units` have been defined and assigned values.

Using the `model.add()` method, an output layer (Dense layer) is added to the model. The output layer is typically used for making predictions or classifications. The `units` parameter here specifies the number of neurons in the output layer, and the `activation` parameter indicates the activation function, with softmax being used in this case. The softmax function maps the inputs to a probability distribution, which is suitable for multi-class classification problems.

Next, the model is compiled using the `model.compile()` method. During compilation, you need to specify the loss function, optimizer, and evaluation metrics. Here, `categorical_crossentropy` is chosen as the loss function, which is suitable for multi-class classification problems. The optimizer selected is Adam optimizer, used to optimize the model's weights. The evaluation metric chosen is accuracy, which measures the model's performance during training and testing.

Finally, the `model.summary()` method is used to print an overview of the model. This method displays information about the model's architecture, output shapes, and the number of parameters, helping to verify if the model has been constructed correctly.

Remember, before running the model, ensure that parameters like `num_classes` have been defined and assigned values. In this piece of code, we proceed with training, evaluation, and saving the model.

Using the `model.fit()` method, the model is trained. This method takes input data `x_train` and target data `y_train`, and trains the model based on the specified parameters. The `epochs` parameter indicates the number of training epochs, the `batch_size` parameter specifies the number of samples per batch, and the `validation_data` parameter represents the validation dataset. Metrics such as loss and accuracy during training will be stored in the `history` object.

Next, the `model.evaluate()` method is used to evaluate the model. This method takes input data `x_test` and target data `y_test`, along with the specified `batch_size` parameter. It computes the model's loss and accuracy on the test set and returns the results, which are stored in the `loss` and `accuracy` variables.

Finally, the `model.save()` method is employed to save the trained model to a designated file. In this case, the model is saved as a file named `vocabulary_model.h5`.

Remember, before running the model training and evaluation, make sure that parameters like `x_train`, `y_train`, `x_val`, `y_val`, `x_test`, and `y_test` are properly prepared.

This code segment uses the matplotlib library to visualize the model's accuracy during the training process.

First, using the `plt.plot()` function, the code plots the training set accuracy and validation set accuracy separately. Here, `history.history['accuracy']` represents the historical record of training set accuracy, and `history.history['val_accuracy']` represents the historical record of validation set accuracy.

Next, the `plt.title()` function is used to add a title to the graph, and the `plt.xlabel()` and `plt.ylabel()` functions are used to set labels for the x-axis and y-axis.

Then, the `plt.legend()` function is used to add a legend, which helps differentiate between the training set and validation set accuracy curves.

Finally, the `plt.show()` function is used to display the plotted graph.

Note that before running this code segment, make sure you have imported the matplotlib.pyplot library and that the `history` variable contains the historical records of accuracy.
