# Supplementary figures and images for: Construction and improvement of English vocabulary learning model integrating spiking neural network and convolutional long short-term memory algorithm
Source: PLoS One. 2024 Mar 22;19(3):e0299425. doi: 10.1371/journal.pone.0299425 (PMC10959372; doi:10.1371/journal.pone.0299425)

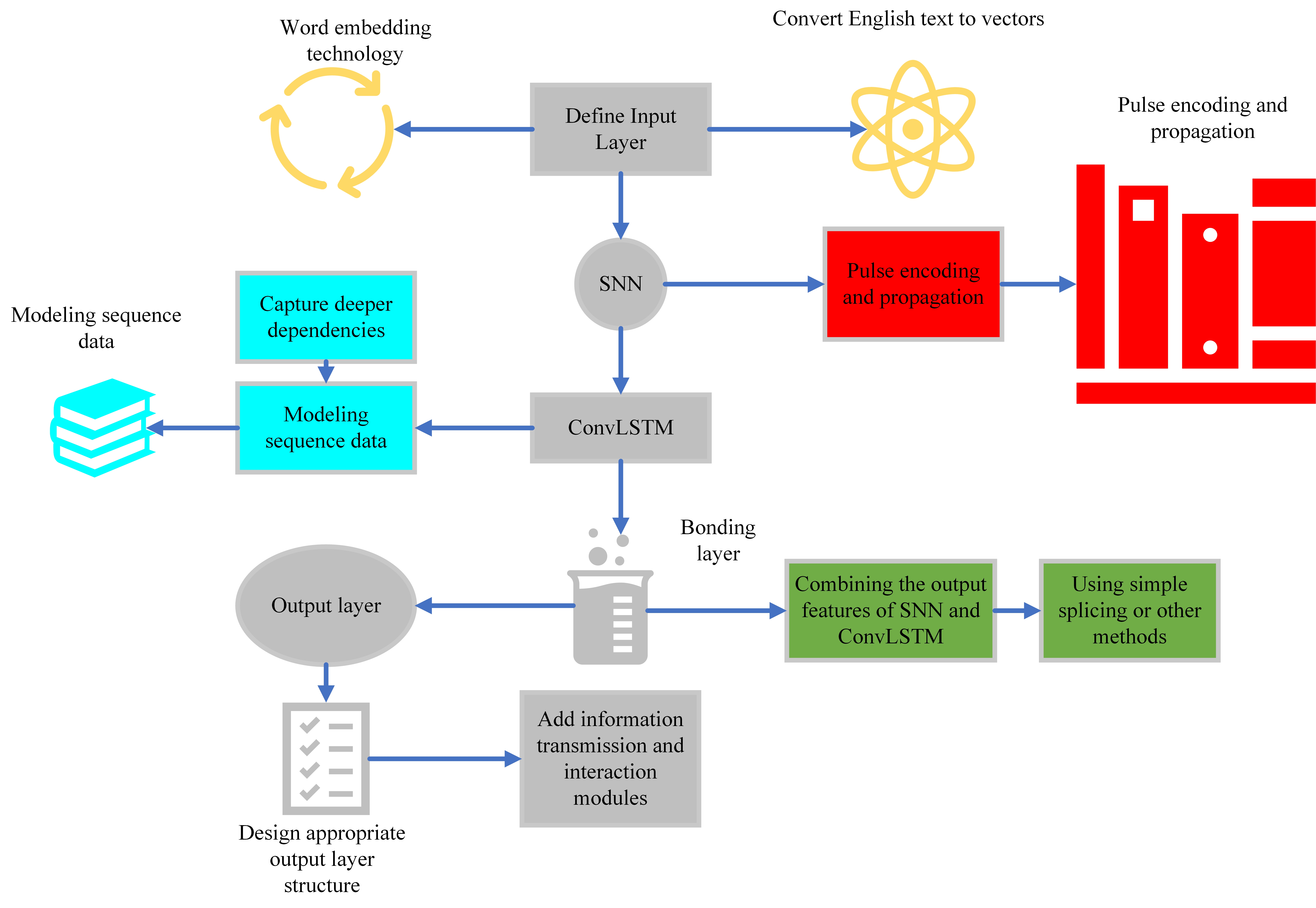

Supplement: S1 Data — (ZIP) [file pone.0299425.s001.zip › ╩2╛▌░n/Figure1.jpg]

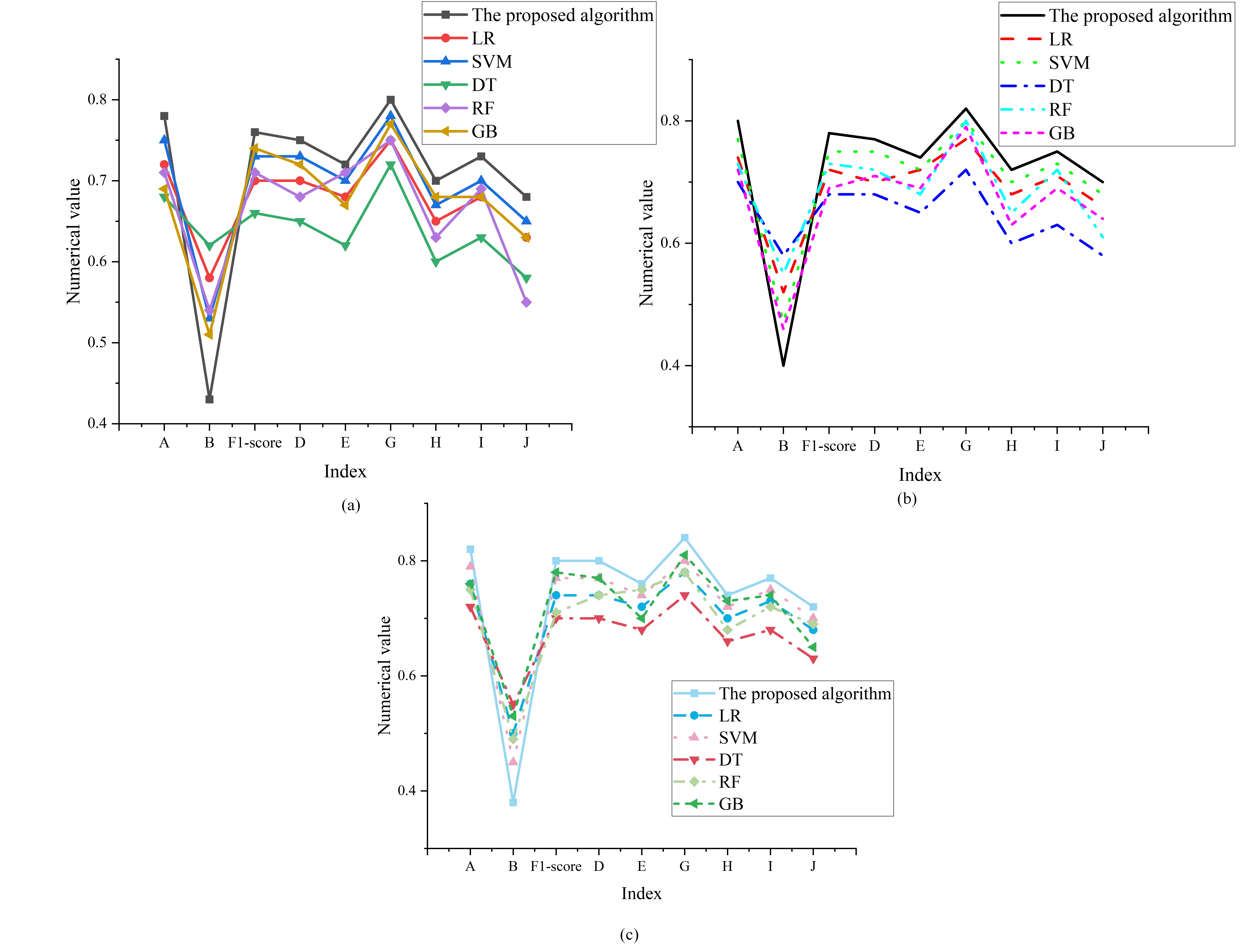

Supplement: S1 Data — (ZIP) [file pone.0299425.s001.zip › ╩2╛▌░n/Figure2.jpg]

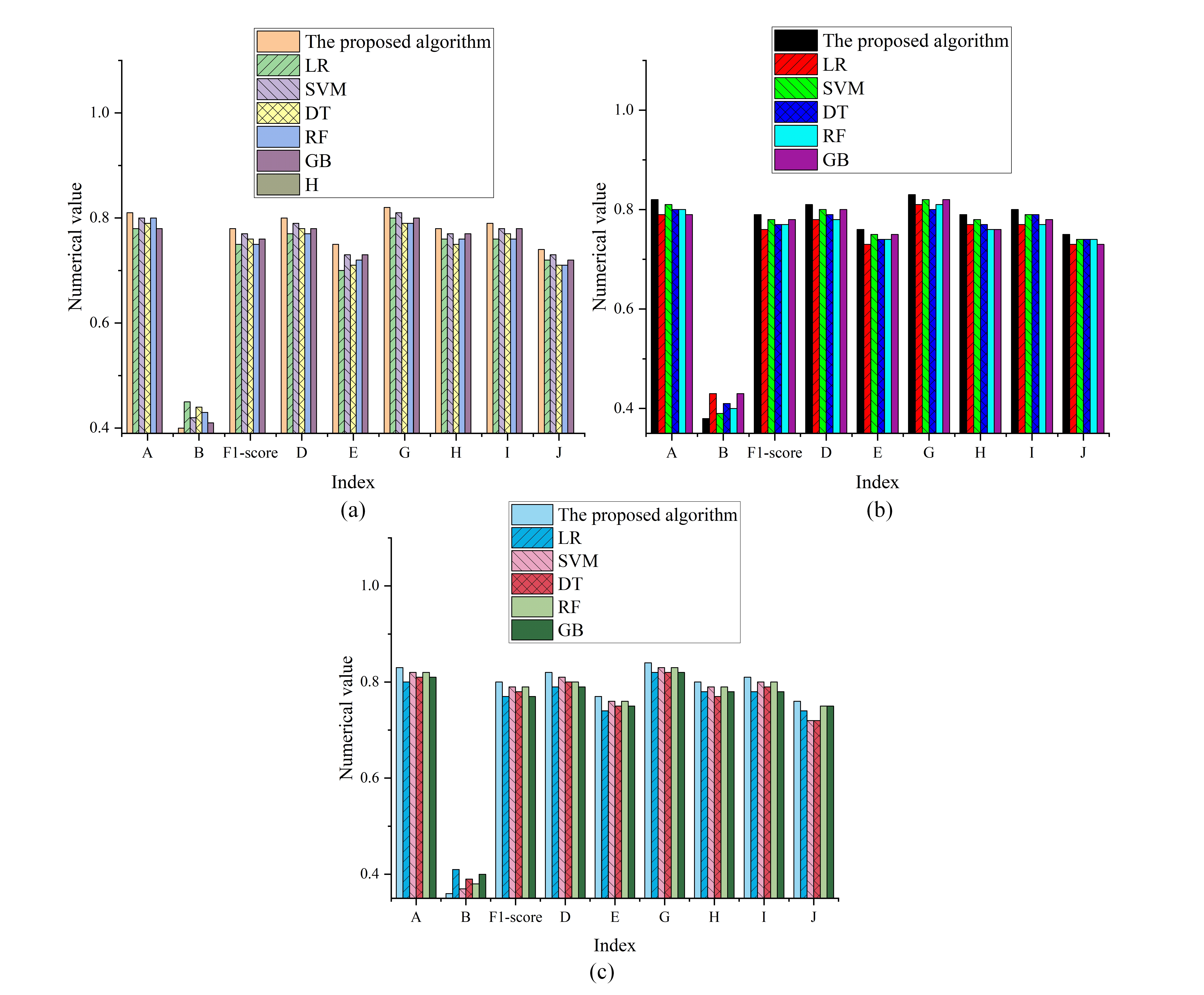

Supplement: S1 Data — (ZIP) [file pone.0299425.s001.zip › ╩2╛▌░n/Figure3.jpg]

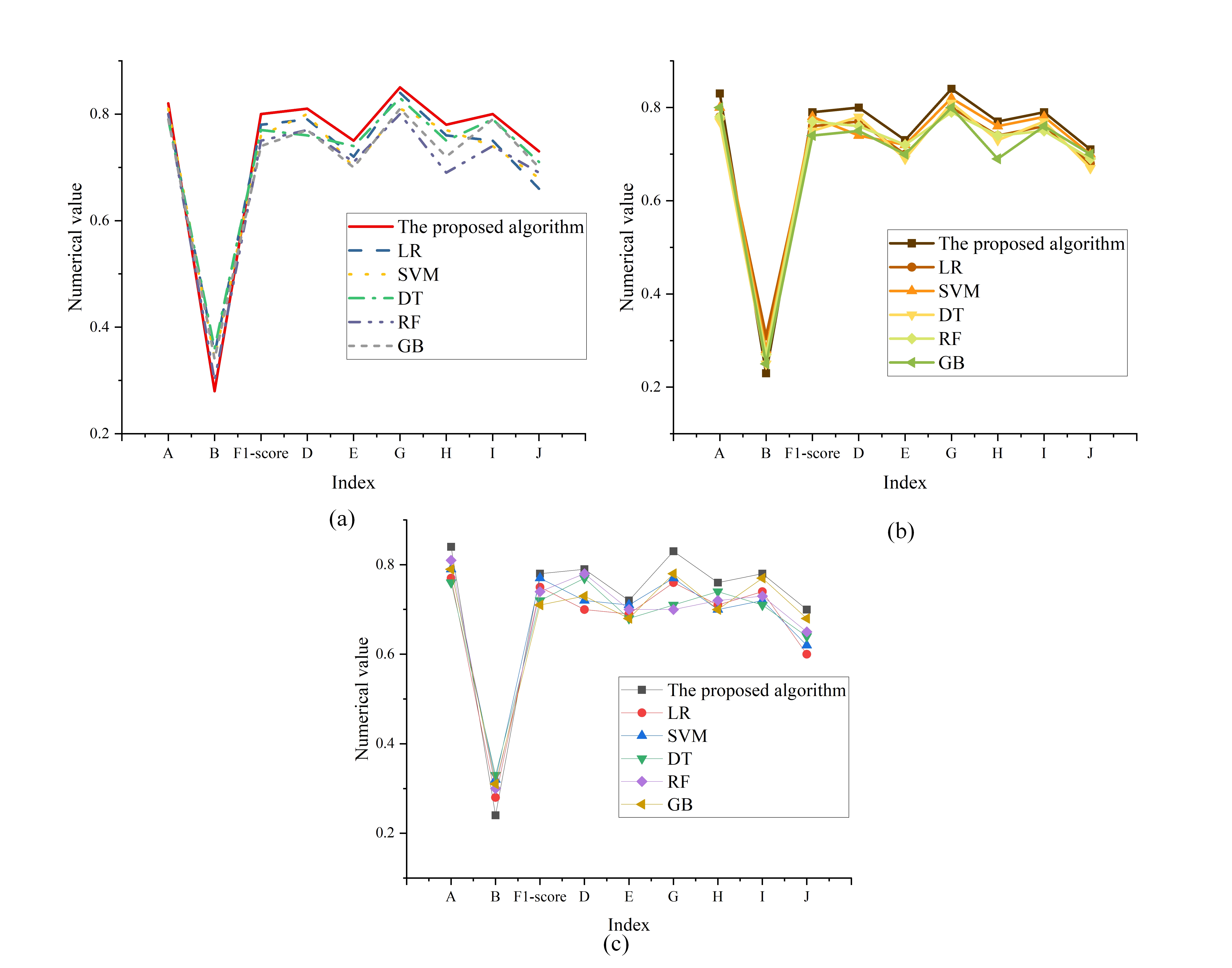

Supplement: S1 Data — (ZIP) [file pone.0299425.s001.zip › ╩2╛▌░n/Figure4.jpg]

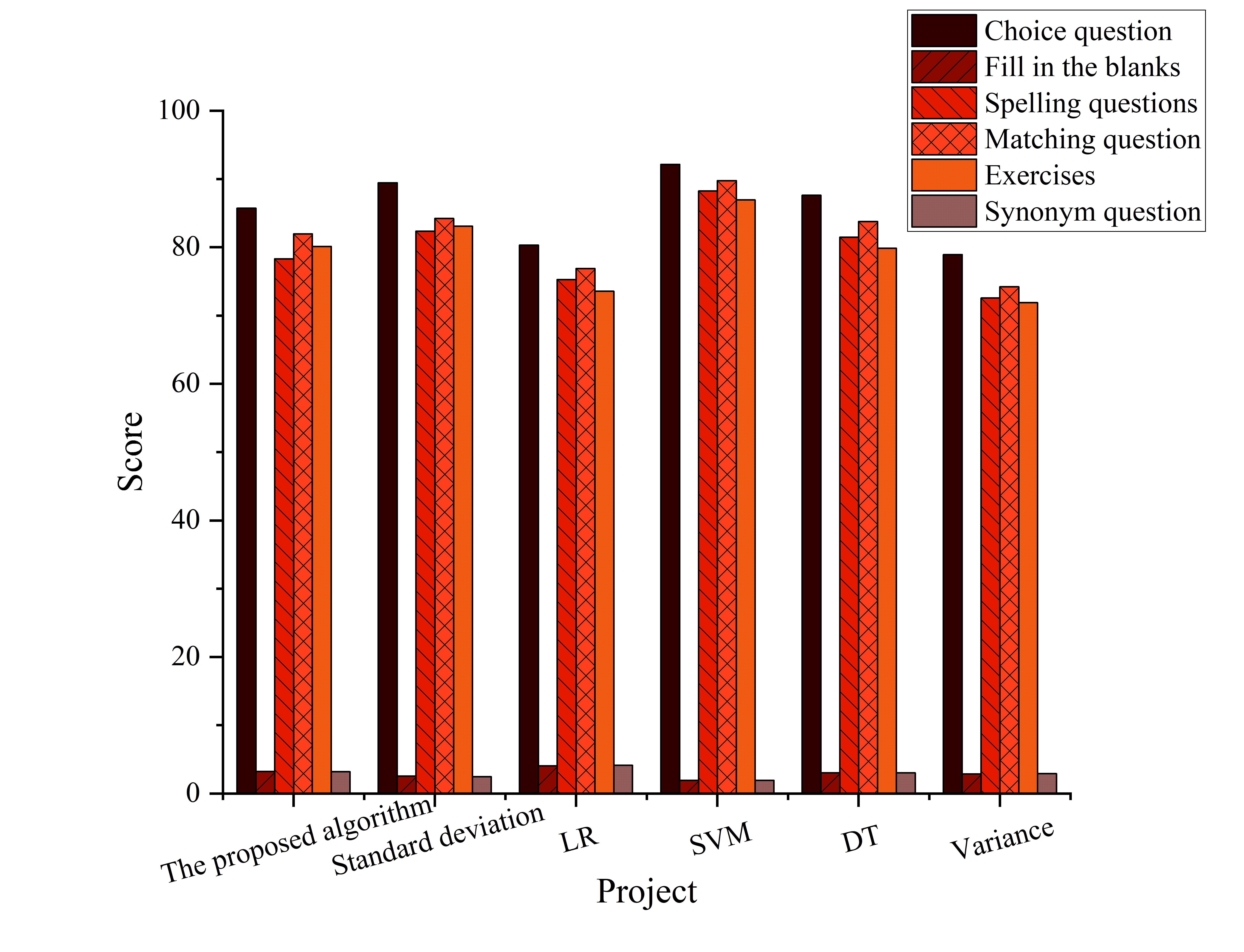

Supplement: S1 Data — (ZIP) [file pone.0299425.s001.zip › ╩2╛▌░n/Figure5.jpg]
